# Charged Residues Flanking the Transmembrane Domain of Two Related Toxin–Antitoxin System Toxins Affect Host Response

**DOI:** 10.3390/toxins13050329

**Published:** 2021-05-01

**Authors:** Andrew Holmes, Jessie Sadlon, Keith Weaver

**Affiliations:** Division of Basic Biomedical Sciences, Sanford School of Medicine, University of South Dakota, Vermillion, SD 57069, USA; Andy.Holmes@coyotes.usd.edu (A.H.); Jessie.Sadlon@SanfordHealth.org (J.S.)

**Keywords:** type I toxin–antitoxin system 2, small protein toxins 3, Fst/Ldr family toxin 4, *Enterococcus faecalis*

## Abstract

A majority of toxins produced by type I toxin–antitoxin (TA-1) systems are small membrane-localized proteins that were initially proposed to kill cells by forming non-specific pores in the cytoplasmic membrane. The examination of the effects of numerous TA-1 systems indicates that this is not the mechanism of action of many of these proteins. *Enterococcus faecalis* produces two toxins of the Fst/Ldr family, one encoded on pheromone-responsive conjugative plasmids (Fst_pAD1_) and the other on the chromosome, Fst_EF0409_. Previous results demonstrated that overexpression of the toxins produced a differential transcriptomic response in *E. faecalis* cells. In this report, we identify the specific amino acid differences between the two toxins responsible for the differential response of a gene highly induced by Fst_pAD1_ but not Fst_EF0409_. In addition, we demonstrate that a transporter protein that is genetically linked to the chromosomal version of the TA-1 system functions to limit the toxicity of the protein.

## 1. Introduction

Bacterial toxin–antitoxin (TA) systems are bipartite modules that encode a toxin that inhibits cellular processes and a cognate antitoxin that neutralizes the toxin. Diverse mechanisms of toxin–antitoxin interaction are used to classify the multiple types of TA modules. Generally, each class differs based on whether the antitoxin is a noncoding RNA or protein and its mechanism of inhibiting the toxin (for recent reviews, see References [1,2,3]). TA systems were first discovered on bacterial plasmids, where they function as post-segregational killing (PSK) or addiction modules that ensure plasmid stability [4,5]. TA modules constitute part of the bacterial mobilome found on other mobile genetic elements (MGE), including transposons, phages, integrons, integrative conjugative elements (ICE) and genomic islands, where they presumably contribute to the evolution of the bacterial genome [6]. In addition to their presence on MGE, TA systems have been found to be ubiquitous on the chromosomes of a wide variety of bacterial species [7]. A multitude of functions have been proposed for chromosomal TA systems, including altruistic suicide, persistence to antibiotic challenge, growth suppression in response to stress, phage inhibition, and biofilm formation, but few have been experimentally demonstrated and some are controversial [2,8,9].

The toxins of type I TA systems (TA-1) are generally small proteins of less than 50 amino acids, whose translation is repressed by a small regulatory RNA [10,11]. These proteins contain a stretch of hydrophobic residues forming a putative transmembrane (TM) domain and some have been demonstrated to be membrane localized [12,13]. Originally identified as plasmid stability loci operating via a PSK mechanism, it was presumed that they functioned by forming non-specific pores and disrupting membrane function [4]. The identification of chromosomal TA-1 systems necessitated a rethinking of their PSK role and detailed examination suggested that lethal pore formation might not adequately describe their function [11,14,15]. Some systems still appear to form pores, but in a manner that only temporarily depolarizes membranes, halting growth and facilitating persistence [16]. In other systems, membrane disruption appears to be a secondary effect or to not happen at all [17,18]. Indeed, it has been suggested that TA toxins in general do not function to kill cells [8,19]. At the same time, there is growing evidence that bacterial cells encode a plethora of small membrane-active proteins apart from TA-1 systems that perform specific functions, such as regulating sugar and ion transport systems [20,21,22,23]. It seems possible, if not likely, that some TA-1 “toxins” have evolved not to be toxins at all, but rather to perform specific cellular functions that only become toxic to the cell when overactive, either due to ectopic production or by disruption of their sRNA repressor. Indeed, in some cases, TA-1 antitoxin sRNA disruption is tolerated by the cell [24].

The *par*_pAD1_ locus encoded by the *Enterococcus faecalis* plasmid pAD1 was the first TA-1 described in Gram-positive bacteria [25,26]. Overexpression of the *par*_pAD1_ toxin, Fst_pAD1_, results in nucleoid condensation, chromosome partitioning, and cell-division defects, followed by increased membrane permeability [17,27]. Bioinformatic analysis revealed that Fst_pAD1_ was the founding member of a subclass of TA-1 toxins widespread among the Firmicutes and Enterobacteriaceae denoted the Fst/Ldr superfamily [28,29,30]. The superfamily signature motif, as defined by Fozo et al. [30], is a highly conserved tryptophan residue flanked by an upstream putative TM domain and a highly charged C-terminus. Curiously, Fst_pAD1_ substitutes valine for the conserved tryptophan indicating that this residue is not required for function. Further analysis defined a conserved P/D/S/TXXXG(C) motif within the TM, where the initial proline, aspartic acid, serine, or threonine residues define four different clades; X is any hydrophobic amino acid; and the terminal cysteine residue is found only in *Staphylococcus* orthologues [13]. NMR structural analysis of Fst_pAD1_ [31] and PepA1 [32] (a member of the Fst/Ldr family in *Staphylococcus aureus*) in membrane mimetics revealed that the TM forms a continuous α-helix across the lipid bi-layer with the C-terminal and N-terminal charged amino acids protruding. In the case of Fst_pAD1_, the longer C-terminus was predicted to extend into the cytoplasm and possibly interact with specific membrane proteins. Surprisingly, while Fst_pAD1_ functions only when produced within cells, PepA1 is capable of lysing both bacterial and eukaryotic cells when added externally [32], suggesting that significant differences in mechanisms of membrane insertion and function may exist within the Fst/Ldr superfamily. However, the molecular details of toxin action and the function of chromosomal loci encoding Fst/Ldr toxins remains obscure.

**Figure 1:**
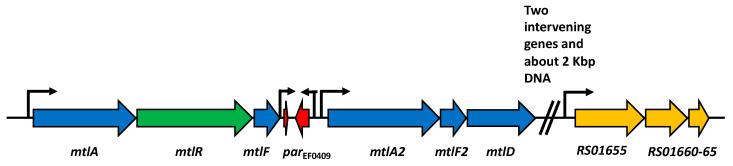
Genome map of *par*_EF0409_ and environs. The two components of the *par*_EF0409_ toxin–antitoxin system are shown in red, the antitoxin on the left and the toxin on the right; *par*_EF0409_ is flanked by paralogous mannitol-type PTS transporter systems (*mtlA-mtlF* and *mtlA2-mtlF2*); *mtlR* encodes a putative positive transcriptional regulator; and *mtlD* mannitol-1-phosphate 5-dehydrogenase. RS01655 encodes a putative efflux protein with the two downstream genes being the other two components. As noted, there are two genes and approximately 2 kbp of DNA between *mtlD* and RS01655. Broken arrows represent putative promoters for the various genes.

In addition to the plasmid-encoded *par*_pAD1_ locus, *E. faecalis* harbors a chromosomally encoded Fst/Ldr toxin in the TA-1 *par*_EF0409_ [28]. Nestled between two paralogous mannitol family phosphotransferase (PTS) sugar transport system gene sets (Figure 1), the *par*_EF0409_ locus is hypothesized to modulate sugar transport [33,34]. A recent study [33] determined that expression of Fst_EF0409_ and Fst_pAD1_ from vector pCIE exhibits shared and distinct effects on the transcription of several *E. faecalis* membrane proteins. For example, expression of both toxins resulted in approximately 32-fold induction of the gene for magnesium transporter MgtA (OG1RF_RS05570) and approximately eight-fold repression of PTS component CelA3 (OG1RF_RS03875). In contrast, the gene most highly induced by Fst_pAD1_, OG1RF_RS02610 (homologous to metal transporting ATPases) was not significantly induced by Fst_EF0409_. Moreover, the gene for an efflux transporter, OG1RF_RS01655, which is located approximately 6 kbp from the Fst_EF0409_ gene (Figure 1), was induced 16-fold by Fst_EF0409_ and only eight-fold by Fst_pAD1._ Given the small size of the peptide toxins and the relatively few amino acid differences, we sought to identify the amino acid determinants of the specificity of induction of OG1RF_RS02610 and OG1RF_RS01655. To do so, we constructed a collection of truncations, domain swaps, and amino acid substitutions and examined their effects on the expression of the aforementioned genes: *mgtA* and *celA3* as controls and OG1RF_RS02610 and OG1RF_RS01655 as differential responders. Using these mutants, we determined the role of the C-terminal tail in toxicity and identified key amino acid residues responsible for the distinct transcriptional response of OG1RF_RS02610 to the two toxins. While we were unable to identify amino acid residues involved in the specificity of expression of OG1RF_RS01655, we demonstrated that this gene limits the toxicity of both toxins.

## 2. Results

### 2.1. Importance of the Charged C-Terminal Tail to Fst Toxicity

Previous results had indicated that the charged C-terminal tail of Fst_pAD1_ was not essential for toxicity [28]. However, these experiments did not allow us to determine the degree of toxicity. Furthermore, recent work with another Fst/Ldr family member, Lpt from *Lactobacillus rhamnosus*, indicated that the charged C-terminus was required for toxicity [35]. To address these issues and determine if the C-terminal tail was involved in differential transcriptomic responses, a series of truncations of both Fst_pAD1_ and Fst_EF0409_ were constructed in the expression vector pCIE (Table 1). A seven amino acid truncation of Fst_pAD1_, which was analogous to the largest truncation mutants showing toxicity in previous experiments [28], detectably slowed cell growth upon maximal induction but less so than wild-type (WT) toxin. Thus, while WT toxin essentially stops growth at these levels of induction, Fst_pAD1_ter7 increased generation time from 0.6 to 0.8 h in uninduced cells and from 1 to 1.4 h in maximally induced cells (Figure S1). So, while the C-terminal tail is not essential, its deletion significantly reduces toxicity. Deletions of two and five amino acids from the C-terminal tails of both Fst_pAD1_ and Fst_EF0409_ had no detectable effects on cell growth at maximal induction and only modest and variable effects at lower induction levels (data not shown).

To investigate potential subtle effects not captured by growth curves and to determine if the C-terminal tail might be involved in the observed differential specificity of Fst_EF0409_ and Fst_pAD1_, we performed qRT-PCR on four genes previously shown to be impacted by toxin induction [33] as described in the Introduction. Genes *mgtA* and *celA3* were used as controls, the former induced by both toxins and the latter repressed, to assess general effects of mutations on gene expression, while genes OG1RF_RS02610 and OG1RF_RS01655 were assessed to determine the mutations effects on specificity. Note we will use the colloquial gene names *mgtA* and *celA3* and just the RS numbers for the other two genes for simplicity of presentation.

**Figure 2:**
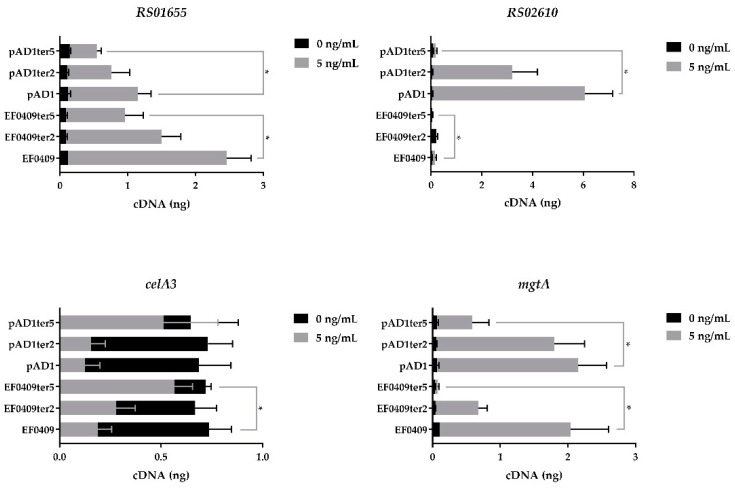
Effects of C-terminal truncations of Fst_pAD1_ and Fst_EF0409_ on expression of responsive genes. Black bars: toxin uninduced. Gray bars: toxin induced with 5ng/mL cCF10. Error bars represent standard error of the mean. Significant differences (*p* < 0.05) indicated as “*”.

Expression of the two and five amino acid truncations resulted in a reduced transcriptional response of all four genes compared to the WT toxins (Figure 2). Uniformly, the effect was greater for the five amino acid truncation than for the two amino acid truncation. These results show that, (1) while the highly charged C-terminal tail is not essential for toxicity, the individual amino acids do affect the response of the host cell and may play a significant role in toxin function; (2) since the truncations affected both control and differentially expressed genes, the C-terminal tail is not solely responsible for the differential effects of Fst_pAD1_ and Fst_EF0409_ on transcriptome response; and (3) loss of the ability to maximally affect expression of the genes examined did not detectably reduce growth inhibition by the toxins.

### 2.2. Identification of the Key Residues for the Differential Response of RS02610

**Figure 3:**
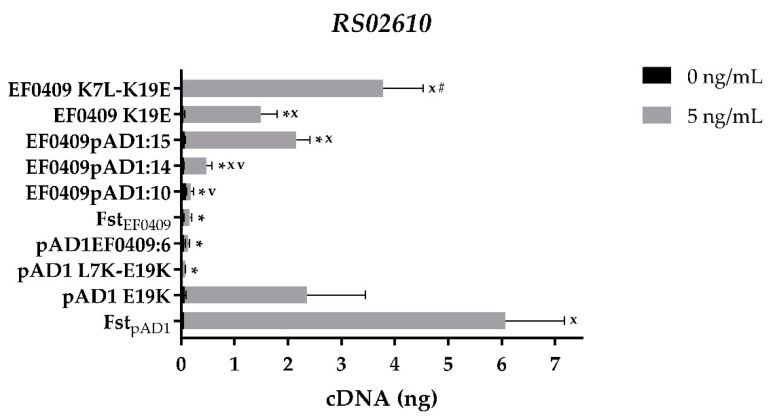
Effect of mutations and swaps on differential expression response of RS02610 to the Fst_pAD1_ and Fst_EF0409_ toxins. Error bars represent standard error of the mean. Amino acid substitution(s) and hybrid mutants were compared to both WT toxins. Significant differences (*p* < 0.05) for mutant samples are shown as follows: “*” relative to Fst_pAD1_; “x” relative to Fst_EF0409_; “#” relative to EF0409 K19E; “v” relative to EF0409pAD1:15.

While the C-terminal truncations reduced the transcriptomic response to toxin expression, they did so across the board and not just to differentially responsive transcripts. To determine which amino acids were responsible for the differential effects, a series of domain swaps between the two toxins were constructed (Table 1). Swapping of the six C-terminal amino acids from Fst_EF0409_ onto Fst_pAD1_ (pAD1EF0409:6) eliminated the differential induction of RS02610 just as truncation of the last five amino acids did (Figure 2 and Figure 3). Similar to the truncation, the amino acid swap retained full toxicity (data not shown) and had reduced effects on the expression of controls *mgtA* and *celA3* (Table S1). The reciprocal swap, EF0409pAD1:8, did not confer the ability to induce RS02610 on Fst_EF0409_ (Table S2). Therefore, the C-terminal tail of Fst_pAD1_ is essential but not sufficient for differential induction of this gene.

To identify other amino acids essential for RS02610 induction, domain swaps were constructed that had progressively more Fst_pAD1_ C-terminal amino acids swapped onto Fst_EF0409_ (Table 1). As shown in Figure 3, a swap of 10 amino acids (EF0409pAD1:10) had no effect on the ability of the toxin to induce RS02610. However, a swap of 14 amino acids (EF0409pAD1:14) increased induction by nearly three-fold, while a swap of 15 amino acids (EF0409pAD1:15) increased induction greater than 13-fold. EF0409pAD1:8 and EF0409pAD1:12 had similar effects as EF0409pAD1:10 and EF0409pAD1:14, respectively (Table S2). These results indicated that (1) the non-consensus V in Fst_pAD1_ does not play a role in the differential induction of RS02610, (2) the S22R23 residues of Fst_pAD1_ may play a moderate role in differential induction, and (3) E19 plays a key role in the differential response.

To determine if the switch at amino acid 19 was sufficient or if other amino acids in the C-terminal tail were required, the single mutant EF0409 K19E was constructed. Induction of RS02610 was significantly higher in response to expression of EF0409 K19E than WT Fst_EF0409_, confirming the importance of this specific residue (Figure 3). Induction with the single mutant was consistently, though not significantly, lower than the 15 amino acid tail swap, however, suggesting that the rest of the tail might have a subtle effect on the response. The effects of the K19E mutation were specific to RS02610 as there was no significant difference in expression of the other three genes examined (data not shown). The reciprocal amino acid change to the Fst_pAD1_ toxin, pAD1 E19K, showed a decrease in expression of RS02610, further supporting a critical role for this residue in differential induction (Figure 3). Again, the effect of the E19K mutation was specific to RS02610 as no significant change was observed in the other three genes (data not shown).

The fact that both EF0409 K19E and pAD1 E19K had intermediate effects on the expression of RS02610 suggested that another amino acid residue(s) might be important for the differential response of the two toxins. Since the TM region is highly conserved between the two toxins, we considered the possibility that the more divergent N-terminal domain might be involved. To test this possibility, the N-terminal seven amino acids from Fst_pAD1_ were swapped onto the EF0409 K19E mutant to create EF0409pAD1N6-K19E. This construct increased induction of RS02610 consistently but not significantly relative to EF0409 K19E (data not shown). Since the N-terminal swapped region removed an Fst_EF0409_ lysine residue that marks the junction with the hydrophobic TM domain and the K19 residue had proven critical in the C-terminal region, we constructed the double mutants EF0409 K7L-K19E and pAD1 L7K-E19K. As shown in Figure 3, the Fst_EF0409_ double mutant increased induction of RS02610 significantly above the single mutant while the Fst_pAD1_ double mutant showed greatly reduced induction of RS02610. The pAD1 L7K-E19K mutant inhibited growth only slightly less than WT Fst_pAD1_ at low levels of expression (1 and 5 ng/mL of cCF10) (Figure S2) and demonstrated similar effects on expression of the other genes examined (Table S1), ruling out a general effect on membrane insertion or other function.

### 2.3. The Differential Response of RS01655 May Relate to Its Function as an Efflux Pump

**Figure 4:**
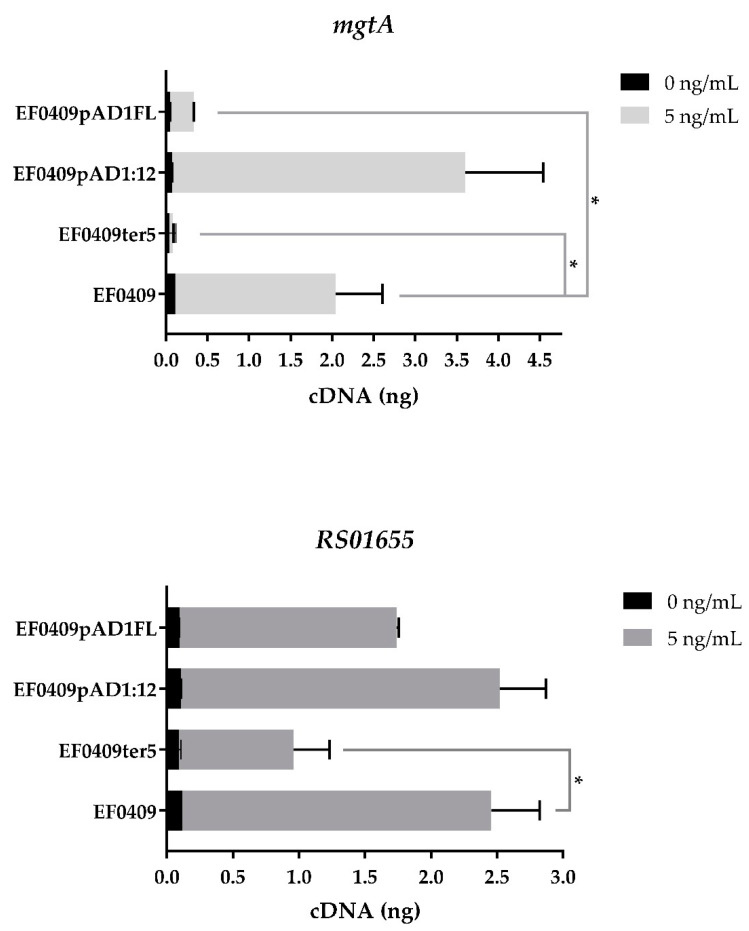
Effects of Fst WT and mutant toxins on RS01655 and *mgtA*. Error bars represent standard error of the mean. Significant differences (*p* < 0.05) are indicated as “*” relative to Fst_EF0409_.

The differential response of RS01655 to the Fst toxins was of interest for two reasons. First, unlike RS02610, expression of the RS01655 transcript is more responsive to Fst_EF0409_ than Fst_pAD1_ ([33] and Figure 2). Second, the gene is genetically closely linked to the *mtlA-par*_EF0409_-*mtlA2* region (Figure 1), suggesting that it may be functionally linked as well. Examination of the various swaps and single base change mutants that impacted RS02610 expression revealed that they did not significantly affect RS01655 expression or consistently alter the ratio of response to the two toxins (data not shown). Furthermore, the effect of C-terminal toxin truncations on RS01655 expression was reduced compared to the other indicator proteins (Figure 2). For example, while EF0409ter5 was still able to induce RS01655 greater than 10-fold, induction of the *mgtA* control dropped from about 20-fold for Fst_EF0409_ to about twofold for the mutant (Figure 4). Additionally, a serendipitous synthesis error led to the construction of a toxin hybrid with an FL mutation at the junction between the Fst_EF0409_ TM domain and the Fst_pAD1_ C-terminal tail (Table 1). This mutation significantly reduced induction of *mgtA* but had only a limited effect on RS01655 (Figure 4). The analogous fusion without the FL mutation, EF0409pAD1:12, induced *mgtA* as well as wild-type Fst_EF0409_ (Figure 4). The robustness of RS01655 induction to the FL and truncation mutations, relative to the other transcripts examined, indicated that it is much less discriminating in its response and may respond predominantly to the hydrophobicity of the proteins.

**Figure 5:**
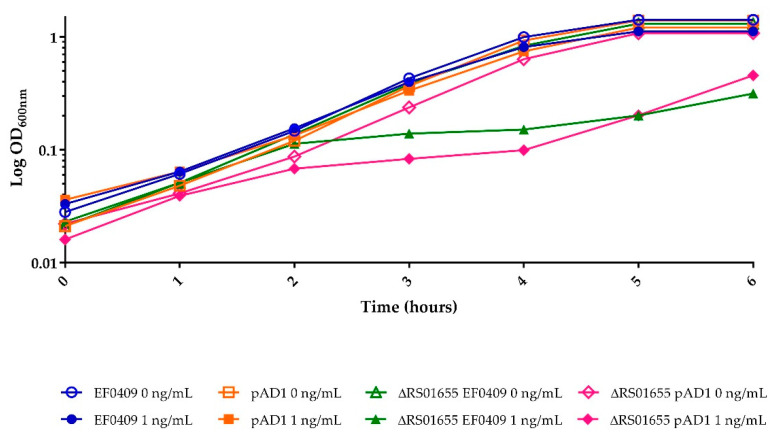
Effect of RS01655 deletion on sensitivity to Fst toxins.

As mentioned above, RS01655 is homologous to efflux transporters. The proximity of its gene to the *par*_EF0409_ locus and its induction by the Fst toxins suggested that it might function to mitigate the effects of toxin expression. To test this hypothesis, an in-frame deletion mutant was constructed within RS01655 by allelic replacement on the chromosome and the effect on toxicity of the two WT Fst toxins was examined. As shown in Figure 5, cell growth was affected by much lower levels of toxin expression in the RS01655 deletion mutant than in WT, with maximal inhibition occurring at 1 ng/mL of inducing pheromone. Similar results were observed with both Fst_EF0409_ and Fst_pAD1_. These results support the hypothesis that the function of RS01655 is to export and thereby mitigate the effects of the Fst toxins, although we cannot rule out indirect effects at this point. Interestingly, the RS01655 mutation did not increase sensitivity of externally added nisin, a lantibiotic unrelated to TA-1 toxins. Inhibition was detectable at nisin concentrations of 1 μg/mL in both WT and deletion strains and did not differ at concentrations up to 8 μg/mL (data not shown).

## 3. Discussion

Previous research determined that even low levels of expression of the two *par*-related toxins had widespread effects on the cellular transcriptome. However, while transcription of some genes was affected similarly in response to both toxins, others showed distinct transcriptional responses [33]. These fine-tuned differences between the transcriptomic effects of Fst_pAD1_ and Fst_EF0409_ expression suggested some manner of target specificity. In order to assess which amino acid residues were responsible for the observed specificity, mutant derivatives of each toxin were constructed and expressed from the pCIE vector as previously described [33]. Progressive deletion of the C-terminal residues of both toxins reduced toxicity as measured both by reduced growth inhibition and reduced effects on transcription of all four indicator genes. Therefore, the charged residues of the C-terminal tail appear to be important for maximal function of the toxin but amino acid differences in the tail between the two toxins are not responsible for specificity of the transcriptomic response.

In contrast to truncations, C-terminal and N-terminal swaps had little effect on expression changes of the controls and no discernable effect on growth inhibition. However, swaps replacing either of the Fst_EF0409_ lysine residues with their Fst_pAD1_ counterparts resulted in the gain of function to induce RS02610, and mutation of both lysines in Fst_EF0409_ K7L-K19E allowed levels of RS02610 induction comparable to those of WT Fst_pAD1_. Conversely, introduction of both lysine residues into the analogous positions in Fst_pAD1_ L7K-E19K, eliminated the ability to induce RS02610, while introduction of a single lysine at E19K had an intermediate effect. While it is possible that the Fst_pAD1_ mutations had non-specific effects, such as altering its ability to insert into the membrane properly, the retention of near WT growth inhibition and effects on control genes argue against this conclusion. Therefore, the results with the double mutants suggest that the positive charges at K7 and K19, flanking the TM domain of Fst_EF0409_ restrict its activity in such a way as to prevent induction of the RS02610 gene. This restriction is relieved by replacing the lysines with the hydrophobic and negatively charged amino acids of Fst_pAD1_. Curiously, a C-terminal tail swap of six amino acids from Fst_EF0409_ to Fst_pAD1_ also eliminated the ability to induce RS02610, suggesting that the amino acids flanking the TM domain may not be the entire story.

**Figure 6:**
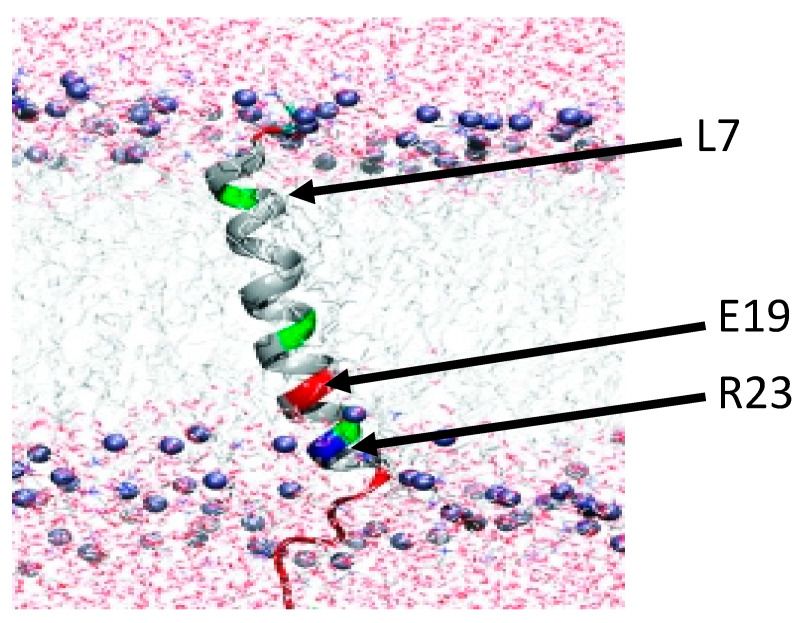
Structural model of Fst_pAD1_ within the bacterial membrane. The N-terminus of the protein is depicted on the external side of the membrane (top) and the highly changed C-terminal tail is depicted extending into the cytoplasm (bottom). Polar residues are indicated in green, positively charged residues in blue, and negatively charged residues in red. Residues relevant to this study are indicated with arrows. Figure is reprinted with permission from Reference [31].

Although this study has identified the amino acid determinants of induction specificity between Fst_pAD1_ and Fst_EF0409_ for RS02610 induction, it is unresolved how these residues interact with the plasma membrane and/or target proteins to produce the differing transcriptional response. We hypothesize that Fst_pAD1_ and Fst_EF0409_ have discrete interactions with plasma membrane components and/or target membrane proteins that are dictated by the differing amino acid residues at positions 7 and 19. Previous research on the solution structure of Fst_pAD1_ (Figure 6) indicated that E19 and R23 form a salt bridge between their side chains and/or establish a connection with the hydrophilic phospholipid head group of the membrane lipids [31]. The formation of a local salt bridge between these two amino acids is likely; a connection between glutamic acid and an arginine residue four positions away is one of the most favorable stabilizing interactions within a helical structure like Fst_pAD1_ [36,37,38]. As salt bridges have been demonstrated to promote protein-protein interfacing and binding of prosthetic groups or cofactors [39], we hypothesize that the E19-R23 connection plays a role in the coordination of Fst_pAD1_ and membrane protein targets. At the corresponding positions, Fst_EF0409_ contains K19 and I23, which would not form a comparable salt bridge. The fine-tuned interactions of Fst_pAD1_ with membrane targets may also be affected by possible extensions of E19 and R23 side chains to the polar head groups of the membrane, as well as the solvation of the L7 residue within the hydrophobic lipid core. These electrostatic, ionic, and/or polar interactions may facilitate the association of Fst_pAD1_ with its respective membrane components, thereby impacting the ability of the toxin to interact with certain targets. In contrast, Fst_EF0409_ may have restricted mobility due to the positively charged K7 and K19 residues that form a clamp with the hydrophilic head groups of the membrane. While it is tempting to suggest that Fst_pAD1_ interacts directly with RS02610, it remains possible that the toxin has indirect effects on membrane structure or another membrane protein that triggers expression of this transporter protein. Future experiments will be necessary to determine the respective target interaction mechanism(s) of Fst_pAD1_ and Fst_EF0409_.

Unlike RS02610, induction of RS01655 was relatively robust to amino acid changes, swaps and even C-terminal deletions, suggesting that induction of its transcription may be responsive to the TM domain itself. Given its homology to efflux transporters and its genetic linkage to the *par*_EF0409_ locus, we considered the possibility that it might function to limit the toxic effects of Fst expression. The fact that mutation of the gene resulted in a dramatic increase in sensitivity to both Fst_EF0409_ and Fst_pAD1_ is supportive of this hypothesis. Therefore, the function of the RS01655 efflux transporter may be to limit the extent of growth suppression under conditions triggering Fst_EF0409_ expression. It is interesting to note that Fst_pAD1_ induces RS01655 significantly less than Fst_EF0409_; perhaps the plasmid-encoded version of the system has evolved to reduce export of its toxin.

It should be noted that, at this time, it is not clear how the expression levels we obtain artificially by induction from the expression vector compare to what the cell would normally experience from the natural loci. Fst_pAD1_ would be expected to be produced only transiently upon plasmid loss, and to our knowledge no one has successfully quantified toxin expression from a PSK system upon loss of its native plasmid. In the case of Fst_EF0409_, the antitoxin sRNA of *par*_EF0409_ is produced in substantial molar excess over the toxin mRNA under all growth conditions examined so far [33]. Therefore, expression of Fst_EF0409_ would require increased transcription of the mRNA and/or decreased stability or transcription of the sRNA antitoxin. Further work will be required to determine under what conditions such changes occur and how much toxin is produced. So, at this time, we cannot rule out the possibility that toxin expression from pCIE, even at the relatively low cCF10 levels used in these experiments, is in excess of what the cell would ever experience. Nevertheless, given the broad range of membrane active small proteins produced by bacteria and by their eukaryotic hosts, we feel that establishing the rules of functionality and specificity of individual amino acid residues is of value in determining their mechanisms of action. The results reported here provide a foundation for designing future experiments to discern the localization of the toxin and what, if any, specific protein targets they interact with.

## 4. Materials and Methods

### 4.1. Bacterial Strains, Media, and Growth Conditions

The *E. faecalis* strain used for all experiments in this study was OG1RF [33,40]. All *E. faecalis* cultures for growth curves and RNA preparation were grown in M9YEG medium [41] with chloramphenicol (Cm) (Sigma-Aldrich, St. Louis, MO, USA) added for plasmid selection where appropriate. Cultures were routinely grown overnight with 25 µg Cm per ml and diluted to one or two percent in fresh medium with 10 µg Cm per mL. Expression from pCIE was accomplished by the addition of the desired concentration of peptide pheromone cCF10 (H-LVTLVFV-OH) from Mimotopes (Clayton, Australia) one hour after dilution, to ensure the cultures were in logarithmic phase. Pheromone was dissolved in dimethylformamide and used at the concentrations indicated in the described experiments. Uninduced strains, labeled as 0 ng/mL, had an equivalent volume of DMF added. Cultures used for RNA purification were grown for one hour after pheromone addition prior to harvest. All liquid cultures were grown at 37 °C with rotary shaking at 25 rpm. *E. faecalis* cultures for electroporation experiments were grown in Todd Hewitt Broth (THB) (Sigma-Aldrich). The *E. coli* strain DH5α (New England Biolabs, Ipswich, MA, USA) was used for sub-cloning from commercially acquired constructs into the pCIE expression vector. All *E. coli* cultures were grown in Luria-Bertani [42] medium. Ampicillin (amp) was used at a concentration of 100 µg/mL for selection of commercial constructs and Cm at 25 µg/mL for selection of pCIE constructs. Liquid cultures were grown at 37 °C with rotary shaking at 250 rpm. Where necessary, solid medium was prepared by addition of 17g agar (Research Products International, Mt. Prospect, IL, USA) per one liter of medium. Plates were grown at 37 °C.

### 4.2. Genetic Manipulations

Mutant constructs encoding Fst_pAD1_ and Fst_EF0409_ derivatives (Table 2) were commercially synthesized and delivered in plasmid pUCminusMCS (Blue Heron Biotech, LLC, Bothell, WA, USA).

Constructs contained flanking *BamH*I and *Sph*I restriction enzyme recognition sites that were used to subclone fragments in pCIE in the proper orientation for expression. All restriction enzymes and DNA ligase were purchased through New England BioLabs or Promega (Madison, WI, USA) and used according to provided protocols. A post-ligation cut was performed with *Sal*I that eliminated any pCIE plasmid without the desired fragment. Ligated DNA was transformed into competent *E. coli* DH5α cells per manufacturer instructions (Invitrogen, Waltham, MA, USA) with selection for pCIE-encoded Cm resistance. Bacterial colonies were then selected and tested for ampicillin resistance to ensure the absence of pUCminusMCS. Plasmid purification from DH5α was performed by using the Quantum Prep plasmid miniprep kit (Qiagen, Germantown, MD, USA) according to their instructions. Mutant inserts were tested for proper base pair length via restriction enzyme digests and agarose gel electrophoresis. Then pCIE constructs with the appropriate restriction pattern were inserted into *E. faecalis* cells by electroporation [43,44]. Freshly electroplated colonies were then assessed for equal sensitivity to the toxin. Plasmid DNA was purified from electroporants via a modified Quantum Prep plasmid miniprep kit protocol [45]. Plasmids showing the expected restriction digest pattern were then sequenced by Eurofins Genomics LLC (Louisville, KY, USA) using the pCIE-EF0409 FWD/REV primers (Table 2) to ensure that no spurious mutations were introduced during the subcloning process.

An in-frame, markerless deletion of gene RS01655 was constructed in OG1RF, using the vector pJH086 [46]. The mutant allele was synthesized by Blue Heron Biotech, LLC, and contained the first 5 and last 5 codons of the RS01655 and approximately 900 base pairs upstream and downstream of the 5′ and 3′ ends of the gene, respectively. The construct was synthesized with *Sph*I and *Sma*I restriction sites at each end, and these enzymes were used to subclone the fragment from the commercially provided pUCminusMCS vector to pJH086. After a post-ligation cut with *BamH*I, plasmid was purified and introduced into competent DH5α cells with selection for Cm and growth at 30 °C. Plasmid was purified, checked for appropriate restriction pattern, and introduced into *E. faecalis* cells as described above with selection at 30 °C. Selection of recombinants was carried out as previously described [47]. Recombinants were screened by colony PCR [44], using primers flanking the desired deletion (Delta01655 FWD and REV Table 2). PCR products showing the appropriate size for the deletion were then sequenced to ensure that no spurious mutations were obtained. Toxin expression plasmids were introduced into this strain by electroporation and induction performed as described above. Sensitivity to nisin was determined by adding nisin (Sigma-Aldrich, St. Louis, MO, USA) after 1 h of growth at concentrations of one, two, four and eight μg/mL.

### 4.3. RNA Purification, Manipulation, and Statistical Methodology

Conduction of RNA purification, manipulation, and qRT-PCR followed the previously established protocol [33]. All samples had a minimum of two biological replicates performed, with key samples having three or more biological replicates. Significant differences in cDNA nanograms for samples induced with 5 ng/mL cCF10 were calculated by utilizing Welch’s t-test, assuming unequal variance with criterion α = 0.05. Fold change was calculated via dividing the induced 5 ng/mL cCF10 sample by its uninduced counterpart.

## Figures and Tables

**Table 1:** Fst toxins with encoded amino acid sequence.

Mutant Fst toxin	Sequence ^a^
Fst_pAD1_	VKDLMSLVIAPIFVGLVLEMISRVLDEEDDSRK
pAD1ter2	VKDLMSLVIAPIFVGLVLEMISRVLDEEDDS
pAD1ter5	VKDLMSLVIAPIFVGLVLEMISRVLDEE
pAD1ter7	VKDLMSLVIAPIFVGLVLEMISRVLD
Fst_EF0409_	MYEIVTKILVPIFVGIVLKLVTIWLEKQNEE
EF0409ter2	MYEIVTKILVPIFVGIVLKLVTIWLEKQN
EF0409ter5	MYEIVTKILVPIFVGIVLKLVTIWLE
pAD1EF0409:6	VKDLMSLVIAPIFVGLVLEMISRVLEKQNEE
EF0409pAD1:8	MYEIVTKILVPIFVGIVLKLVTIWLDEEDDSRK
EF0409pAD1:10	MYEIVTKILVPIFVGIVLKLVTIVLDEEDDSRK
EF0409pAD1:12	MYEIVTKILVPIFVGIVLKLVSRVLDEEDDSRK
EF0409pAD1:14	MYEIVTKILVPIFVGIVLKMISRVLDEEDDSRK
EF0409pAD1:15	MYEIVTKILVPIFVGIVLEMISRVLDEEDDSRK
EF0409 K19E	MYEIVTKILVPIFVGIVLELVTIWLEKQNEE
pAD1 E19K	VKDLMSLVIAPIFVGLVLKMISRVLDEEDDSRK
EF0409pAD1N6-K19E	MKDLMSLILVPIFVGIVLELVTIWLEKQNEE
EF0409 K7L-K19E	MYEIVTLILVPIFVGIVLELVTIWLEKQNEE
pAD1 L7K-E19K	VKDLMSKVIAPIFVGLVLKMISRVLDEEDDSRK
EF0409pAD1FL	MYEIVTKILVPIFVGIVLKLVFLVLDEEDDSRK

^a^ Fst_pAD1_ residues are shown in black, Fst_EF0409_ residues in red, and erroneously inserted residues in blue.

**Table 2:** Primer names and designated sequences.

Primer	Sequence
pCIE-EF0409 FWD	GTATACAGTTCATGTATATGTTCCC
pCIE-EF0409 REV	TGTGATGCACCTCCTTTC
RS02610 FWD	CAGATGACGGCTCAATTCAAAC
RS02610 REV	CAGCGGTACTTCCTTCAATCA
RS01655 FWD	GCACGATGTCTGGTGATGAT
RS01655 REV	CTTCGCTCCTAAATCCGCTAAG
*mgtA* FWD	AAAGGTGCGGTTGAAGAAATG
*mgtA* REV	TGACGCAGTGTCTCTGTTAAG
*celA3* FWD	AGAAGATCGTGGCATGGAAG
*celA3* REV	TGAAACGAACTTGTGGACCTAA
GyrB FWD	ACCAACACCGTGCAAGCC
GyrB REV	CAAGCCAAAACAGGTCGCC
Delta01655 FWD	GGGTCCTTCTGTGTGTGTAAATA
Delta01655 REV	GTCCACTCGGCTAAACGTATAAT

## Data Availability

All data relevant to the central findings of this study are included within the article and supplementary material.

## Supplementary Materials

The following are available online at https://www.mdpi.com/article/10.3390/toxins13050329/s1. Table S1: Comparison of effects of Fst_pAD1_ and derivatives on gene expression. Table S2: Effect of mutations and swaps on differential expression response of RS02610 to the Fst_pAD1_ and FstEF0409 toxins. Figure S1: Effect of C-terminal truncation of seven amino acids from Fst_pAD1_ on growth inhibition; Figure S2: Effect of double mutants on growth inhibition.
