# Supplementary material for: Charged Residues Flanking the Transmembrane Domain of Two Related Toxin–Antitoxin System Toxins Affect Host Response
Source: Toxins (Basel). 2021 May 1;13(5):329. doi: 10.3390/toxins13050329 (PMC8147318; doi:10.3390/toxins13050329)
Supplement: Supplementary file 1 [file toxins-13-00329-s001.zip › toxins-1164826-supplementary.pdf]

Supplementary Materials: Charged Residues Flanking the Transmembrane Domain of Two Related Toxin-Antitoxin System Toxins Affect Host Response

Andrew Holmes, Jessie Sadlon and Keith Weaver

Table 1. Comparison of effects of Fst_{pAD1} and derivatives on gene expression. SEM is depicted within the parentheses.

Fst Toxin	Avg. ng cDNA (0ng/mL cCF10)	Avg. ng cDNA (5ng/mL) cCF10	Fold Change
	RS01655		
Fst_{pAD1}	0.121 (±0.034)	1.150 (±0.193)	9.497
pAD1 L7K-E19K	0.031 (±0.001)	0.902 (±0.134)	29.129
pAD1EF0409:6	0.116 (±0.033)	0.626 (±0.037)	5.394
	RS02610		
Fst_{pAD1}	0.054 (±0.016)	6.073 (±1.098)	111.749
pAD1 L7K-E19K	0.016 (±0.002)	0.075 (±0.009)	4.841
pAD1EF0409:6	0.057 (±0.018)	0.134 (±0.027)	2.370
	celA3		
Fst_{pAD1}	0.685 (±0.159)	0.125 (±0.074)	-5.495
pAD1 L7K-E19K	0.319 (±0.017)	0.062 (±0.008)	-5.171
pAD1EF0409:6	0.839 (±0.113)	0.443 (±0.152)	-1.892
	mgtA		
Fst_{pAD1}	0.067 (±0.027)	2.154 (±0.420)	32.051
pAD1 L7K-E19K	0.014 (±0.009)	0.354 (±0.108)	21.880
pAD1EF0409:6	0.057 (±0.012)	0.346 (±0.018)	6.070

Table 2. Effect of mutations and swaps on differential expression response of RS02610 to the Fst_{pAD1} and Fst_{EF0409} toxins. SEM is depicted within the parentheses.

Mutant Fst toxin	Avg. ng cDNA (0ng/mL cCF10)	Avg. ng cDNA (5ng/mL) cCF10	Significance [a]
Fst_{pAD1}	0.054 (±0.016)	6.073 (±1.098)	x
pAD1ter2	0.057 (±0.011)	3.207 (±0.996)	
pAD1ter5	0.079 (±0.012)	0.195 (±0.036)	*
Fst_{EF0409}	0.042 (±0.009)	0.164 (±0.040)	*
pAD1EF0409:6	0.057 (±0.018)	0.134 (±0.027)	*
EF0409pAD1:8	0.064 (±0.009)	0.186 (±0.034)	*, v
EF0409pAD1:10	0.097 (±0.003)	0.182 (±0.050)	*, v
EF0409pAD1:12	0.047 (±0.004)	0.401 (±0.051)	*, v
EF0409pAD1:14	0.056 (±0.003)	0.472 (±0.103)	*, x, v
EF0409pAD1:15	0.070 (±0.008)	2.162 (±0.253)	*, x
EF0409 K19E	0.049 (±0.014)	1.491 (±0.301)	*, x
pAD1 E19K	0.065 (±0.023)	2.360 (±1.090)	
EF0409 K7L-K19E	0.020 (±0.002)	3.779 (±0.752)	x, #
pAD1 L7K-E19K	0.016 (±0.002)	0.075 (±0.009)	*

[a] Significant differences ($p < 0.05$) for mutant samples are shown as: "*" relative to Fst_{pAD1}; "x" relative to Fst_{EF0409}; "#" relative to EF0409 K19E; "v" relative to EF0409pAD1:15.

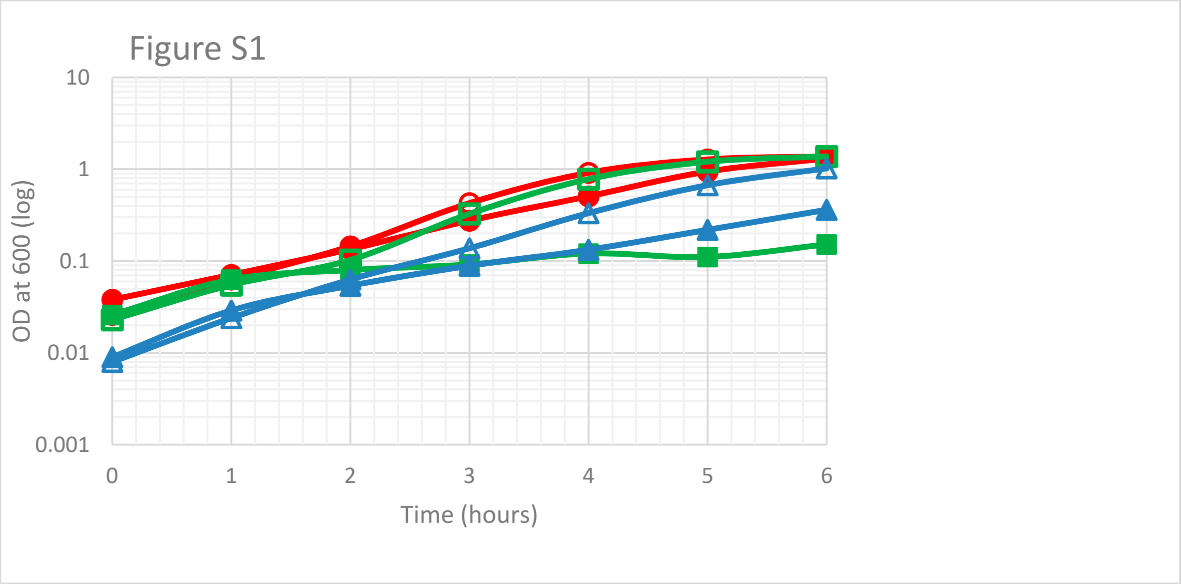

Figure S1. Effect of C-terminal truncation of seven amino acids from Fst_{pAD1} on growth inhibition. Results are from two independently isolated transformants of pCIE::pAD1ter7 into OG1RF compared to OG1RF with vector alone. Open symbols are without cCF10 induction, closed symbols are induced with 50 ng/mL cCF10. Green=OG1RF (pCIE::Fst_{pAD1}). Red=OG1RF (pCIE::pAD1ter7) isolate 1. Blue=OG1RF (pCIE::pAD1ter7) isolate 2.

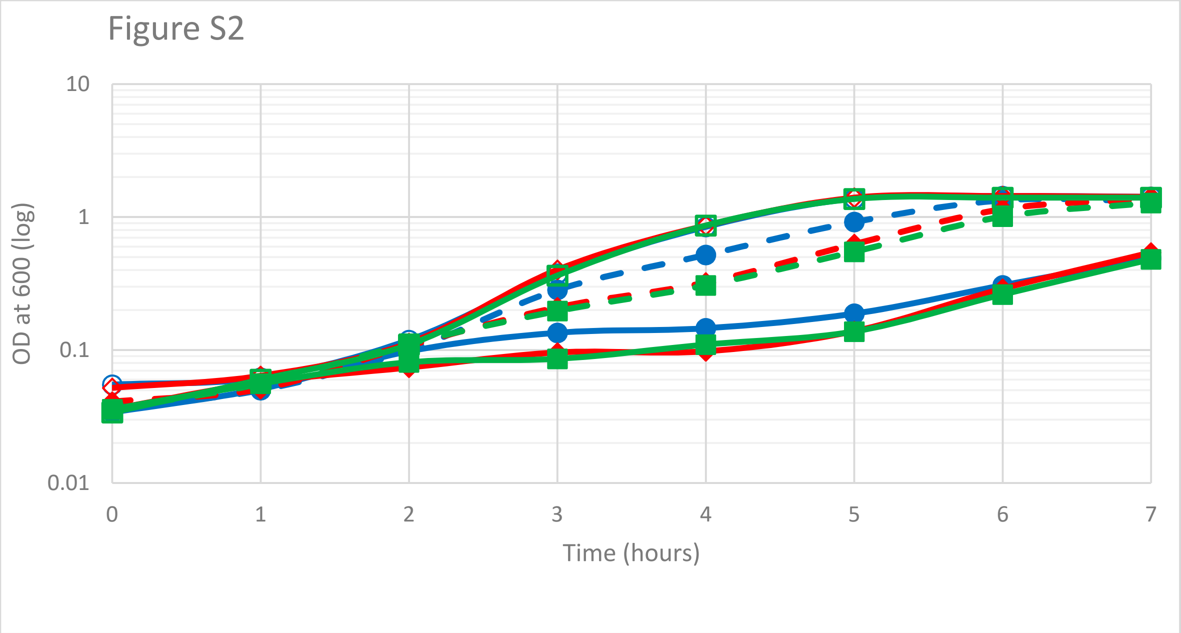

Figure S2. Effect of double mutants on growth inhibition. Open symbols are without cCF10 induction, closed symbols with dashed line are induced with 1 ng/mL cCF10, closed symbols with solid line are induced with 5 ng/mL cCF10. Green=OG1RF (pCIE::Fst$_{pAD1}$). Red=OG1RF (pCIE::EF0409 K7L-K19E). Blue=OG1RF (pCIE::pAD1 L7K-E19K).
